# Interspinous Process Implantation for the Treatment of Neurogenic Intermittent Claudication

**DOI:** 10.5812/aapm.5173

**Published:** 2012-07-10

**Authors:** Nasser Heyrani, Elizabeth Picinic Norheim, Yeelan Elaine Ku, Arya Nick Shamie

**Affiliations:** 1 David Geffen School of Medicine, University of California, Los Angeles, USA; 2 Department of Orthopaedic Surgery, Harbor-UCLA Medical Center, Los Angeles, USA; 3 Department of Orthopaedic Surgery, Santa Monica-UCLA Medical Center, Los Angeles, USA

**Keywords:** Spinal Stenosis, Intermittent Claudication, Magnetic Resonance Imaging

## Abstract

**Background:**

Lumbar spinal stenosis (LSS) is a disabling medical condition in which narrowing of the spinal canal compresses the spinal cord and nerves causing a condition called neurogenic intermittent claudication (NIC). Decompressive spine surgery is the standard of care for patients who fail to improve with conservative management. However, oftentimes, patients who suffer from LSS are elderly individuals with multiple co-morbidities who cannot withstand the risks of decompressive surgery. X-Stop, a novel and minimally invasive FDA approved interspinous process implant, has come into the scene as an alternative to decompressive surgery, and can be inserted under local anesthetic with minimal blood loss.

**Objectives:**

Despite its growing support in medical literature as an effective and conservative treatment of NIC, X-Stop remains a fairly new form of treatment. The aim of this study is to assess the clinical efficacy of its use.

**Patients and Methods:**

Fifty consecutive patients with at least two-year follow-up had a confirmed diagnosis of NIC secondary to LSS by computed tomography or magnetic resonance imaging (MRI) and subsequently received an X-Stop implant. Subjects’ ages ranged from 64 to 95 with a mean age of 79, while the gender distribution comprised of 23 males and 27 females. Zurich Claudication Questionnaire (ZCQ) was used to assess patient outcome measures in three domains: physical function (PF), patient satisfaction (PS), and symptom severity (SS). The visual analog scale (VAS) was used to assess trends in pain with a scale from 0–10, with 0 defined as “pain-free” and 10 designated as “the worst pain imaginable”.

**Results:**

Compared to pre-op scores, PF, SS, and VAS scores for back, buttock and leg pain had a significant mean decrease at 6, 12, 24 months post-op (P < 0.05). Based on the ZCQ and VAS scores, a success rate of 79% (27.34), 78% (30.38) and 74% (17.23) were achieved at six months, 12 months, and 24 months respectively.

**Conclusions:**

X-Stop is a safe and effective treatment for NIC that provides marked relief of symptoms with sustained beneficial outcomes at up to two years of follow-up. In addition, X-Stop permits implantation under local anesthetic with minimal blood loss”.

## 1. Background

Lumbar spinal stenosis (LSS) is a common spinal disorder that typically affects patients over 50 years of age with an estimated 8–11% incidence in the United States (1). As the “baby boomers” age, an estimated 2.4 million Americans will be affected by LSS by 2021 (2). The adjusted rate of lumbar stenosis surgery per 100.000 medicare beneficiaries was 137.4 in 2002 and 135.5 in 2007 (2); these numbers are expected to double in the coming years due to the increased numbers of older adults (2). Verbiest was the first to describe the clinical presentations of neurogenic intermittent claudication (NIC) (3), a condition secondary to LSS. The characteristic symptoms include numbness, pain, and weakness in the buttocks and the lower extremities, which are exacerbated upon extension and alleviated with flexion of the lumbar spine (4–6). Pathologic narrowing of the spinal canal due to LSS is aggravated upon standing, which reduces the cross-sectional area of the neural foramina and spinal canal; while sitting or flexing the spine will relieve symptoms from expansion of the spinal canal (4). Initially, patients are treated with a regimen of non-invasive therapies, which include non-steroidal anti-inflammatory drugs, physical therapy, modifications of daily physical activities, and epidural injections (7, 8). Patients who do not respond to non-operative therapy are historically directed to undergo decompressive surgery, by removal of the structural components of the vertebrae responsible for impinging the cauda equina in order to alleviate NIC (9). X-Stop, a novel and less invasive surgical treatment for NIC, has been previously described (10, 11). The major benefits of X-Stop implementation are its ability to be placed under local anesthetic and minimal blood loss. X-Stop maintains the spinal segments in a slightly flexed and distracted posture and limits pathologic extension (12). Cadaveric X-Stop studies have demonstrated implanted segments with significantly increased canal area, sub articular diameter, and foraminal width as compared to prior to implantation (13, 14). The X-Stop was approved by the FDA in November 2005 (15), and as of October 2006 the centers for medicare and medicaid services have approved a special add-on payment (16).

## 2. Objectives

Despite its growing support in medical literature as an effective and conservative treatment of NIC (7, 11, 17, 18), X-Stop remains a fairly new form of treatment, and additional studies are necessary to show its clinical efficacy and to further define its indications for optimal use. The aim of this retrospective case series is to assess the efficacy of X-Stop surgery up to two years post-surgery.

## 3. Patients and Methods

A retrospective case series was performed at our institution from January 2006 to January 2009. The study was approved by the institutional review board Human Subjects Committee at the University of California, Los Angeles.

### 3.1. Patient Selection

Inclusion criteria were as follows. All patients were required to be greater than 50 years of age. Patients must have attempted and failed at least six months of conservative therapy, such as epidural steroid injection, oral steroids, Nonsteroidal Anti-inflammatory Drugs (NSAIDS), analgesics, physical therapy, and/or spinal manipulation. Symptoms required included leg/buttock/groin pain with or without back pain that was exacerbated by lumbar extension and relieved in flexion. If the back pain was also present it must have been partially relieved when the patient flexed the lumbar spine. All subjects carried a diagnosis of NIC due to lumbar stenosis at one or two lumbar levels that were confirmed by X-ray, MRI or CT. Subjects were all considered surgical candidates with a disease severity justifying that X-Stop IPD placement. In addition, all subjects had a baseline score of > 2.0 in the Physical Function (PF) domain of the ZCQ, which is consistent with FDA approved X-Stop indication for use. Lastly, subjects were required to be capable of walking at least 50 feet. Exclusion criteria were as follows. Subjects with unremitting pain in any spinal position or axial back pain only without leg/buttock/groin pain were not included. Subjects were also excluded with significant instability of the lumbar spine (e.g., spondylolisthesis greater than Grade 1), significant scoliosis (Cobb angle is greater than 25 degrees), degenerative neurologic disease, an ankylosed segment at the affected level(s), a history of spinous process fracture or pars interarticularis fracture, a fixed motor deficit or known peripheral neuropathy, cauda equina syndrome, spinal or systemic infection, or mass lesions (e.g. disc herniations, synovial cysts, spinal tumors). In addition, patients were also excluded for symptoms consistent with vascular claudication, a history of immunologic suppression or having had received 7.5 milligrams prednisone (or equivalent) daily for more than six months immediately prior to enrollment, and a history of bleeding disorder or an active systemic disease such as HIV, hepatitis, etc. Lastly patients with a known allergy to the implant materials, including titanium or titanium alloy and polyetheretherketone, were unable to participate in the study.

### 3.2. Surgical Procedure

All patients received X-Stop implementation under local anesthetic by the same senior surgeon. The type and factory name of the instrument are X STOP Interspinous Process Decompression (IPD) manufactured by Kyphon (Sunnyvale, California.) After IV sedation was administered, patients were placed on a radiolucent Jackson Table in lateral decubitus position, left side up. Patients were encouraged to actively flex their lumbar spine as much as possible with the knee/chest position to passively open the interspinous interval. The patient’s lumbar area was prepped and draped in the standard fashion using an adhesive shower curtain drape. After localization of the appropriate level(s) using fluoroscopic imaging, the skin and the subfascial layers were anesthetized with local anesthetic injection. A 2–5 cm incision was made at midline and exposure was made down to the fascia. Two separate Para central facial incisions were made overlying the appropriate interspinous intervals. Para spinal muscle elevation was accomplished down to the lamina. Self-retaining retractors were used to maintain exposure of the interspinous spaces. As per the X-Stop technique guide, dilators and sizers were used to measure the interspinous space and the appropriate sized implants were implanted gently and the wing inserter was finally used to secure the implant. Wound was irrigated and suctioned dry and two separate facial closures were used to reapproximate the facial incisions prior to standard closure of the subcutaneous and skin layers.

### 3.3. Patient Characteristics

50 consecutive patients with at least two-year follow-up had a confirmed diagnosis of NIC secondary to LSS by computed tomography or magnetic resonance imaging (MRI) and subsequently received an X-Stop implant. Subjects’ ages ranged from 64 to 95 with a mean age of 79, while the gender distribution comprised of 23 males and 27 females. 23 patients had preexisting grade 1 spondylolisthesis prior to surgery and 27 had no preexisting spondylolisthesis. A total of 75 interspinous process implants were placed in 50 patients with 25 patients receiving placement at a single level and 25 receiving an implant at two levels. Overall, one implant was placed at the L1–L2 level, nine were placed at the L2–L3 level, 29 were placed at the L3–L4 level and 36 were placed at the L4–L5 level.

### 3.4. Patient Outcome Assessment

Zurich Claudication Questionnaire (ZCQ) has been shown to be an adequate tool for assessing patient outcome measures in three domains: PF, symptom severity (SS), and the post-operatively obtained patient satisfaction (PS) (19, 20). Both the PF and PS domains are graded from one to four, while SS is from one to five, with one equating best outcome, such as being “very satisfied” in PS and experiencing no physical activity restrictions in the PF domain. As all of the participants were from the elderly population, many patients had other medical complications that were unrelated to NIC and thus, were asked to focus on the NIC-related symptoms in assessing their SS domain as best as possible. The success criteria for X-Stop were based on the mean ZCQ scores in each of the three domains. A score of 2.5 or less in the PS domain and an improvement of at least 0.5 in each of the remaining two domains qualified a treatment as being successful (20, 21). The visual analog scale (VAS) was used to assess trends in pain with a scale from 1-10, with 1 defined as “pain-free” and 10 designated as “the worst pain imaginable.” At each point in time, VAS scores were assessed separately for the back, buttocks, and lower extremities. A six-point improvement on VAS qualified the treatment as being successful. Participants were administered the ZCQ and VAS via telephone. Both the ZCQ and VAS scores were obtained in 40 patients at the six month post-op period, 34 patients at the one-year post-op period, and 26 patients at the two-year post-op period. All of the statistical analysis for this study was completed by an independent statistical lab at the UCLA Department of Biostatistics. Mean comparisons were obtained and analyzed via a parametric repeated measure analysis of variance (RM ANOVA). A *P* value of < 0.05 was considered significant. Informed consent was obtained from each patient included in the study and the ethical guidelines set forth by the University of California, Los Angeles Institutional Review Board were strictly adhered to.

## 4. Results

The average blood loss of the surgery was: 36 cc [range 5 to 70 cc]. Total operative time was 112 minutes [range 56 to 192 minutes].

### 4.1. ZCQ Score

**Figure 1:**
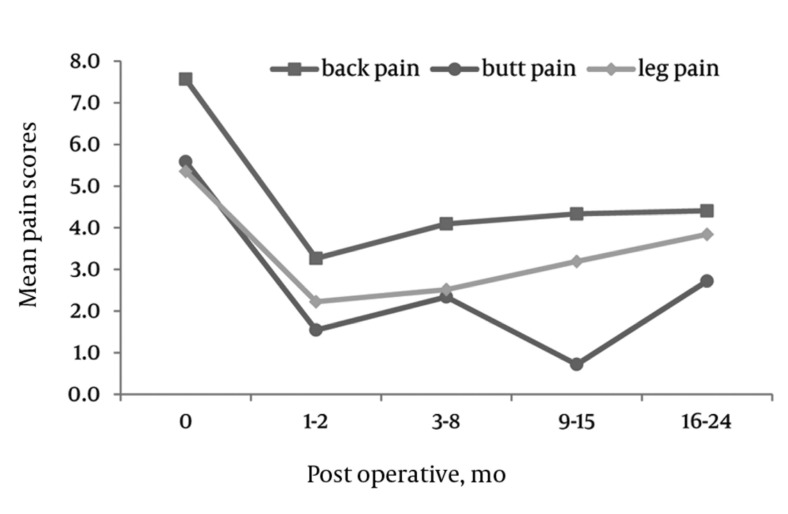
Visual Analog Scale (VAS)

**Figure 2:**
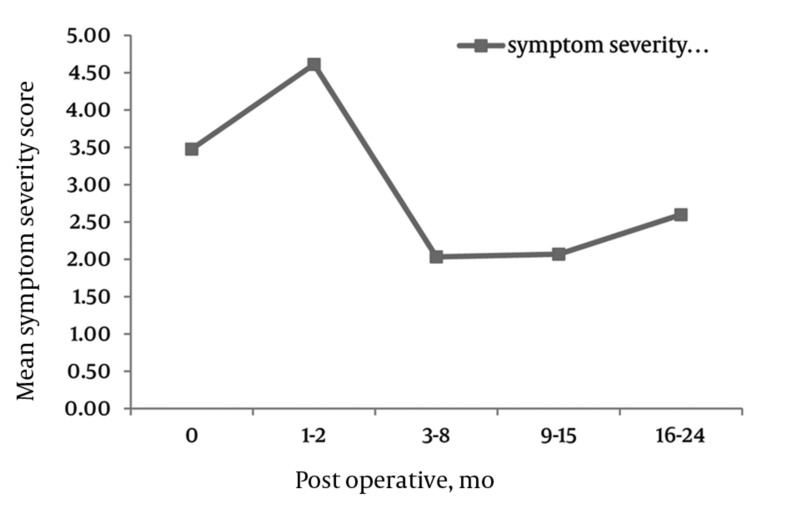
Symptom Severity Scale

**Figure 3:**
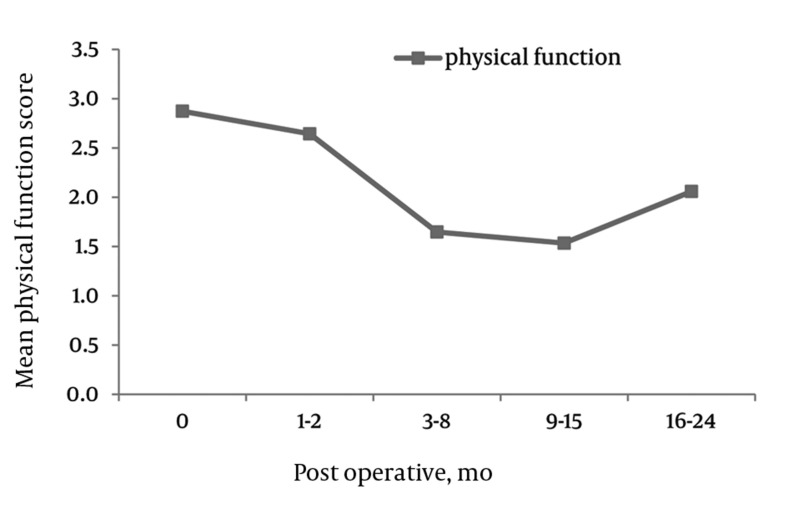
Physical Function (PF) Scale

**Figure 4:**
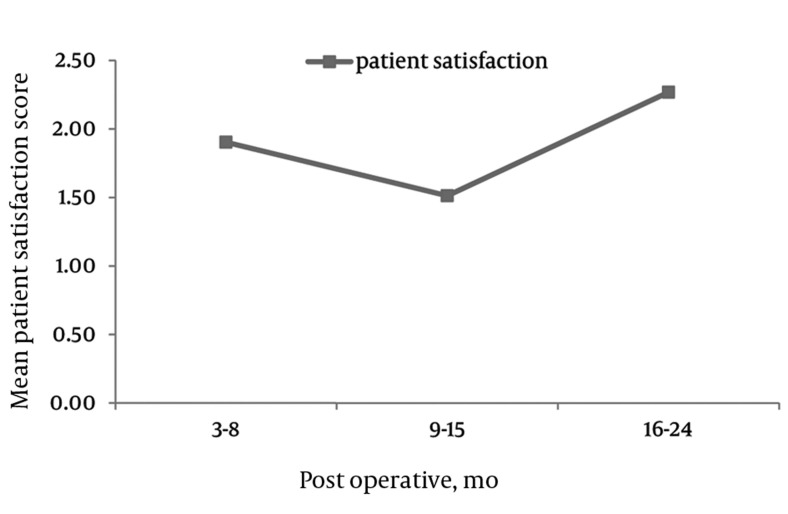
Patient Satisfaction Scores Remained Significantly Low at 6, 12, and 24 Months Post-Operatively

There were significant decreases across all three domains of SS, physical function and PS in the ZCQ (*Figures 1-4*). SS: The mean ZCQ pre-operative SS score was 3.6 (SD = 0.7). The mean SS values significantly dropped to 1.9 (SD = 0.9), 1.6 (SD = 0.9) and 2.1 (SD = 1.0) at 6, 12, and 24 months post-operatively, respectively (*Figure 2*).

PF, The mean ZCQ pre-operative SS score was 3.0 (SD = 0.8). The mean PF values significantly dropped to 1.6 (SD = 0.8), 1.3 (SD = 0.7) and 1.2 (SD = 0.9) at 6, 12, and 24 months post-operatively, respectively (*Figure 3*).

PS, The mean post-operative PS scores were 1.8 (SD = 0.9), 1.4 (SD = 1.2), and 1.8 (SD = 0.8) at 6, 12, and 24 months postoperatively (*Figure 4*). Patients with preexisting grade 1 spondylolisthesis showed significantly greater improvements in all three domains of the ZCQ when compared to patients without spondylolisthesis at two year post-surgery interval: SS 2.6 (SD = 0.8) spondy. *vs.* 2.6 (1.1) no spondy, *P* = 0.0032; PF 1.3 (0.61) spondy *vs.* 2.1 (1.18) no spondy, *P* = 0.0289; PS 1.26 (0.46) spondy *vs.* 2.27 (1.14) no spondy, *P* = 0.0132.

### 4.2. VAS Pain Scale

There were significant decreases in VAS scales for back, buttocks, and leg pain at 6, 12, and 24 months compared to pre-operative values (*Figure 1*).

BACK: The mean VAS pre-operative back pain score was 7.7 (SD = 2.1). The mean pain values significantly dropped to 3.35 (SD = 2.9) 3.35 (SD = 3.2) and 3.25 (SD = 2.9) at 6, 12, and 24 months post-operatively, respectively.

BUTTOCKS: The mean VAS pre-operative buttocks pain score was 6.3 (SD = 3.4). The mean pain values significantly dropped to 2.3 (SD = 2.9), 2.0 (SD = 3.0) and 2.2 (SD = 3.2) at 6, 12, and 24 months post-operatively, respectively.

LEGS: The mean VAS pre-operative leg pain score was 6.1 (SD = 3.6). The mean pain values significantly dropped to 2.5 (SD = 3.0), 2.5 (SD = 3.0) and 3.1 (SD = 3.6) at 6, 12, and 24 months post-operatively, respectively.

### 4.3. Safety/Complications

In all 50 of the patients, there were no intra-operative complications related to the X-Stop device. The implant was removed in 4(8%) patients for failure of the implant to resolve symptoms. For those who the implant was removed, three underwent laminectomy with decompression +/- fusion. There were no post-operative fractures of the spinous processes, implant dislodgement, or infectious wound complications. 24 patients were lost to follow-up. Four were deceased for reasons unrelated to the surgery.

## 5. Discussion

Throughout the medical literature, there is clear evidence that flexion of the spine alleviates symptoms of NIC while extension exacerbates it (1, 3–6). Furthermore, radiological studies have demonstrated that flexion of the spine expands the dimension of the spinal canal to consequently relieve symptoms (4, 22). Given these findings, X-STOP and other minimally invasive interspinous process implants were designed to address NIC by maintaining some flexion, but more importantly distracting the lamina and the posterior disc and, therefore, counteracting symptomatic extension of the spine.

X-Stop is one of the only interspinous process implants that has FDA approval and has been studied by several groups. In 2000, an FDA investigational trial analyzed the efficacy of X-Stop compared to non-operative treatment consisting of physical therapy, analgesics, anti-inflammatory medications and physical therapy in 191 patients using the same ZCQ success criteria we utilized in our study. At one year post-surgery, the success rates in the X-Stop and non-operative treatment groups were 59% and 12%, respectively. Similarly, the PS rates were 73.1% in the X-Stop group compared 35.9% in the non-operative group at the two-year post-surgery period. A major limitation of this clinical trial was the inclusion criteria of at least six months of failed prior conservative non-operative therapy. These inclusion criteria meant patients relegated to the non-operative group had already failed conservative treatment measures for at least six months. The four year follow-up of 18 patients from this same patient pool using the Oswestry Disability Index (ODI) found a 78% (14/18) success rate (23). Again, these findings are limited by a follow-up of only 18 patients despite the enrollment of 200 patients in the initial FDA investigational trial.

In a multi-center prospective study, Zuckerman *et al.* demonstrated at one and two year follow-up that 59% of patients undergoing X-STOP treatment were significantly improved in the ZCQ scores, as compared to the 12% of patients who were provided non-operative management. Kuchta and colleagues reported the two-year follow-up of 175 patients, with an average of 69 years, who had been treated with X-Stop for symptomatic LSS (24). VAS (back, buttocks, and legs) ODI scores were analyzed pre-operatively and post-operatively at six weeks, six months, 12 months, and 24 months. The mean VAS score was significantly reduced from 61.2% to 39.0% at six weeks post-operatively and also 39.0% at the 24 month post-surgery. Similarly, ODI scores were significantly reduced from 32.6% pre-operatively to 22.7% at six weeks and 20.3% at 24 months post-operatively. The X-Stop implant was removed in eight of the 175 patients due to unsatisfactory results with subsequent microsurgical decompression following X-Stop removal. The two-year post-operative reductions in VAS and ZCQ scores reported in our study are consistent with the reductions in VAS and ODI reported in the Kuchta study. This comparison is notable given our patients had an overall mean age (79 years compared to 69 years).

Although, high-grade spondylolisthesis is a contraindication to interspinous spacer placement, there is limited data regarding the clinical outcome of patients with grade 1 spondylolisthesis treated with X-Stop compared to those without spondylolisthesis prior to surgery. A randomized control trial by Anderson *et al.* (17) found significantly better ZCQ and SF-36 scores in patients with pre-existing grade 1 spondylolisthesis that underwent X-Stop Implantation compared to patients treated conservatively. However, this study did not compare clinical outcomes of patients with and without spondylolisthesis following X-Stop implantation. In our study, patients with preexisting grade 1 spondylolisthesis showed significantly greater improvement in VAS and ZCQ scores compared to those without spondylolisthesis. To our knowledge, our study is the first to report statistically significant improvements in clinical outcomes in patients with grade 1 spondylolisthesis compared to those without spondylolisthesis at two years post-surgery.

In our study, we were able to show that X-Stop is a safe and effective treatment for NIC in patients with LSS. The interspinous process implant provided marked relief of symptoms with sustained beneficial outcomes at up to two years of follow-up. The minimally invasive device was implanted under local anesthetic and the procedure had minimal blood loss in all 50 patients. In addition, the minimally invasive implantation permitted subsequent decompression if necessary for patients who failed to have resolution of their symptoms.

Due to the retrospective nature of the patients’ pre-operative symptoms, the study is subject to inherent bias and confounding variables. In addition, although we had one of the largest case series reported for X-Stop, our series can be confounded by selection bias in that we did not have randomization to non-surgical, X-Stop, and/or decompression treatment arms. Lastly we had a significant amount of individuals lost to follow-up despite repeated attempts to contact patients. We therefore cannot make any judgments on the outcomes of these patients.

With the growing elderly population, LSS will continue to be a leading cause of spinal surgery in the future. X-Stop, an interspinous process implant, can significantly improve patients’ NIC symptoms with minimal surgical risks especially in the frequently encountered elderly patient with LSS accompanied by multiple medical co-morbidities. Further studies with longer follow-up and direct comparison of X-Stop versus laminectomy are underway.
